# Photodynamic diagnosis with methyl-5-aminolevulinate in squamous intraepithelial lesions of the vulva: Experimental research

**DOI:** 10.1371/journal.pone.0196753

**Published:** 2018-05-09

**Authors:** Lea Leufflen, Aurelie Francois, Julia Salleron, Catherine Barlier, Gilles Dolivet, Frederic Marchal, Lina Bezdetnaya

**Affiliations:** 1 Institut de Cancérologie de Lorraine, Nancy, France; 2 Université de Lorraine, CNRS, CRAN, Nancy, France; 3 Université de Lorraine, CHRU-Nancy, Institut de Cancérologie de Lorraine, Nancy, France; Massachusetts General Hospital, UNITED STATES

## Abstract

The incidence of the High-grade Squamous Intraepithelial Lesion of the vulva, formerly vulvar intra-epithelial neoplasia is progressively increasing. Today, an early detection and a precise localization of vulvar lesions are still problematic issues, due to the lack of accuracy of the available diagnostic tool. A new approach is the photodynamic diagnosis based on the fluorescence detection of protoporphyrin IX (PpIX) in cancer cells after topical application of a cream of methyl amino-levulinic acid. This study aimed to evaluate the effectiveness of photodiagnosis in order to discriminate the intensity of PpIX fluorescence between vulvar tumor and healthy skin. After topical application of the cream, the fluorescence on xenografted A431 tumor and adjacent skin was non-invasively measured with optical fiber. The tumor to skin fluorescence ratios were 1.38 and 1.41 at respectively 3h and 6h after application, which were significantly higher compared to those observed before application. PpIX accumulation at different depths of the tumor was investigated by spectrofluorimetry after PpIX chemical extraction from tumor sections at 3h and 6h post-application. It was noticed at both application times that the concentration of PpIX within the tumor progressively decreased. However PpIX fluorescence was always detectable up to 2.5 mm, a depth equivalent to more than three quarters of the tumor. The tumor to exposed skin ratios of PpIX fluorescence showed a good selectivity up to1mm depth at 3h post-application and up to 1.5mm at 6h post-m-ALA. Thus, the photodynamic diagnosis using *in vivo* topical methyl amino-levulinic acid appears to be a promising way to detect the intraepithelial lesions of the vulva. Our results open the possibility for implementation of topical methyl amino-levulinic acid in clinical settings for recognition of vulvar high-grade squamous intraepithelial lesions.

## Introduction

Vulvar premalignant lesions induced by HPV are currently grouped under the term "Vulvar High-grade Squamous Intraepithelial Lesion" (vulvar HSIL) formerly known as vulvar intraepithelial neoplasia (VIN usual type) [1]. Due to the risk of recurrence, vulvar HPV-induced HSIL requires conservative treatment whenever possible [1–3]. However, if occult cancer is suspected despite a biopsy of HSIL, wide local excision should be performed. An early detection and precise localization of vulvar HSIL are still problematic issues. The diagnosis is limited to the visual inspection during gynecological examination with confirmation by a punch or small local incision biopsies [4], that may not be representative of tumor stage and size. Interpretation of relief or pigmentation anomalies remains operator dependent. If the lesions are not clearly delineated in women with persistent symptoms, colposcopy may be useful in determining the extent of disease. However, this examination may fail since local inflammation and pruritus could mask the HSIL lesions. Therefore, the risk to underestimate invasive cancer must be considered before treatment [5].

A relatively new, non-invasive and reliable diagnosis technique termed PhotoDynamic Diagnosis (PDD) has been proposed as an interesting complement to clinical examination. PDD is based on the detection of fluorescence emitted by a photosensitizer selectively accumulated in pathological tissues.

Topical 5-aminolevulinate (5-ALA) or its methyl (m-ALA) or hexyl (h-ALA) derivatives are the precursors of the endogenous photosensitizer, protoporphyrin IX (PpIX) and they are increasingly used in PDD and photodynamic therapy (PDT) of cancerous or pre-cancerous diseases, especially in dermatology and urology [6, 7]. Few reports about the use of 5-ALA based PDD for diagnosis of vulvar lesions can be found in the available literature [8, 9]. The most exhaustive clinical study, conducted in 79 women with two different concentrations of 5-ALA, demonstrated a greater sensitivity along with a specificity comparable to colposcopy. This study also showed evidence of the PDD efficiency in the detection of the early stages of vulvar cancer or pre-cancerous lesions. Albeit these encouraging results, preclinical studies are missing to allow the use of PDD as an organized routine vulvar HSIL screening. Further studies are required to evaluate the optimal conditions for the improvement of PDD-based vulvar HSIL specificity and sensitivity.

Ester derivatives of 5-ALA present several advantages over the parent molecule, as they are more stable, they produce a higher amount of PpIX, a better selectivity [10, 11] and they allow an improved fluorescence-assessed delimitation at the site of application. The *in vivo* results observed in pre-clinical models demonstrated that topical application on normal murine skin of m-ALA in cream or bioadhesive patches resulted in high levels of PpIX. Moreover the PpIX induced fluorescence was limited to the site of application [12, 13]. The authors suggested it would be suitable to apply different m-ALA formulations to moist areas such as lower female reproductive tract. Unfortunately, no further development of m-ALA-based PDD for vulvar HSIL in pre-clinical models was disclosed, although this step is essential prior to proposing vulvar HSIL PDD in clinical settings.

The present study aimed to develop in pre-clinical models a reliable photodynamic diagnosis of vulvar HSIL based on topical applications of m-ALA.

## Materials and methods

### Cell culture

A431 cell line, a long-established squamous cell carcinoma of the vulva, was purchased from ATCC^®^ CRL-1555 TM. A431 cells were cultured in phenol-free RPMI medium (Invitrogen, Cergy-Pontoise, France) supplemented with 9% (v.v^-1^) heat-inactivated fetal calf serum (PAN Biotech GmbH, Aidenbach, Germany), 1% (v.v^-1^) penicillin (10 000 IU) streptomycin (10 000 mg.ml^-1^) and 1% (v.v^-1^) glutamin 200.10^-3^M (Invitrogen). Cells were maintained in a humidified incubator (5% (v.v^-1^) CO_2_ in air) at 37°C.

### Animal model

All experiments were performed in accordance with the European Union animal care guidelines and were carried out in an establishment approved by the appropriate authority. The animal project registered under the number 1801–201509171807542 received a favorable opinion from the Ethics Committee and was definitely approved by the French Higher Education and Research Minister. Female NMRI-Foxn1nu/Foxn1nu mice (Janvier Labs, Saint Berthevin, France) aged 7 weeks, with a body weight of 18-25g were used. Five mice were housed per cage, in day/night cycle of 12h at 22–24°C and 55±10% humidity. All procedures involving animals were performed under general anesthesia with inhaled isoflurane (Abbott Laboratories, Abbot Park, IL, USA) using a Univentor 400 anesthesia unit (Genestil, Royaucourt, France). Exponentially growing A431 cells (5 x 10^6^) in 50 μl of 5% glucose water were injected subcutaneously in the right flank. Cells were injected in a close proximity to the skin to achieve maximally superficial tumors. When the tumor volume reached approximately 100 mm^3^ and before any sign of inflammatory skin ulceration, the mice were taken into experiments. For histological examination, animals were sacrificed by cervical dislocation under 5% isoflurane anesthesia, tumor was removed and fixed in 4% formaldehyde 4%. Paraffin sections were further performed before staining with hematoxylin-eosin-safran (HES).

### m-ALA application protocol

In this study, topical application of m-ALA (168 mg/g Metvixia^®^ Galderma, Switzerland) associated with 2% dimethyl sulfoxide was used to enhance the transcutaneous cream penetration [14].

A topical application of 0.25 g of m-ALA cream was performed in mice on the surface of the tumor and adjacent skin for 3 and 6 hours under 2% isoflurane anesthesia. The application of m-ALA on both sites (tumor and adjacent skin) was conducted in the same mouse in order to reduce the number of animals. A flexible mask adapted to the animal was fixed to the flank by a transparent adhesive dressing and perforated with two holes (1 cm diameter). After topical m-ALA application, the flexible mask was covered with another occlusive patch to allow optimal skin penetration. Right after drug application, the animals were placed under dim light conditions to avoid cutaneous photosensitivity. During experiments, animals were regularly observed and no signs of suffering were detected.

### Fluorescence spectroscopy of PpIX after topical m-ALA application

Noninvasive, real-time measurements of PpIX fluorescence (PDT fluorometer V. 1.2 Biolitec, Jena, Germany) in tumors and adjacent skin in mice (n = 10) were performed before, at 3h and 6h after removal of residual cream. Excitation was provided by a light-emitting diode (LED) at a wavelength of 405 nm. The fluorescence spectrum of PpIX was recorded with the optical fiber (200 microns outer diameter) between 600 and 700 nm by a simple contact of the optical fiber perpendicularly to tested tissues. The fluorescence intensity was considered at its maximal value corresponding to λ = 635 nm. At least five measurements of fluorescence were performed for each mouse on tumor and adjacent skin at each tested time (0h, 3h and 6h). A reliability study of the measurements had shown a good consistency of the mean when at least 4 measurements were performed. This analysis was based on the Intraclass Correlation Coefficient (ICC) [15]. The means of these measurements were used to calculate the ratio between tumor fluorescence and exposed skin fluorescence. The measurements of unexposed adjacent skin, away from sites covered with cream, were performed at 3h and 6h post-application serving as internal controls.

### Quantification of PpIX concentration in tissues

In order to study PpIX accumulation in tumor and healthy skin, mice (n ≥ 6) were sacrificed at the end of m-ALA application by cervical dislocation under 5% isoflurane anesthesia. Samples of unexposed adjacent skin, adjacent m-ALA treated skin and tumor were removed, weighted, frozen and embedded into Tissue-Tek^®^ O.C.T. compound before to be cut into sections of 50μm. To obtain a detectable signal of fluorescence, 5 consecutive slices were mixed together (250μm). To solubilize these samples, a volume of Solvable^™^ (Perkin Elmer, USA) as a function of tissues sections weight was added to tissues and placed at 50°C for 2h. At the end of incubation, the absorbance value at 633nm in each sample was adjusted, if necessary, to be < 0.1 to avoid nonlinear effects. The fluorescence signal of PpIX was measured with Perkin-Elmer LS 55 spectrometer (Perkin-Elmer, Beaconsfield, UK). The concentration of PpIX was normalized to mg of tissue based on a standard calibration curve, performed in control tumor or skin tissues.

### Statistical analysis

The fluorescence intensities in the tumor, adjacent skin and its ratios at each time interval were assessed using box-plot analysis. Box plot displayed the variations of tumor and skin fluorescence as well as fluorescence ratio at different conditions. Box plot analysis illustrates the mean, median, min, max and upper and lower quartiles.

Mixed models were used to compare the non-invasive fluorescence measurements in adjacent skin and tumor tissues in order to take into account the correlation between the repeated measures for each mouse. The fixed-effects models were the application time and skin type (adjacent skin *vs*. tumor) and the random-effect model was the mouse. The post-hoc analyses were performed with the Bonferroni correction. For the fluorescence ratio, the fixed-effect was application time only. For the analysis of fluorescence as a function of tumor depth, a mixed model was also performed. Statistical analysis was carried out using SAS software, version 9.2 (SAS Institute, Cary, NC 25513). Significant level was set at 0.05.

## Results

### Histological analysis of xenografted tumors

**Fig 1:**
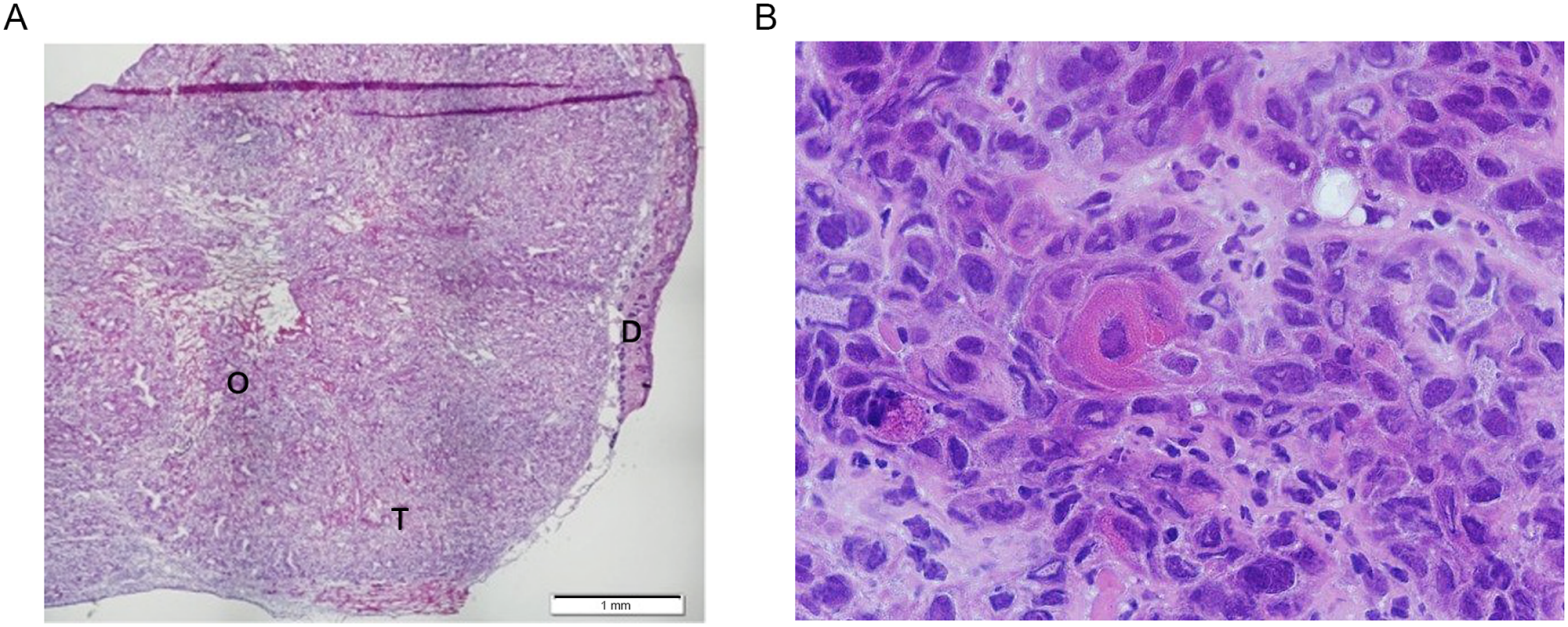
Histological characterization of human A431 subcutaneous tumors in nude mice. Subcutaneous tumor (T) (x4) developed at D7 after inoculation of A431 (histological section 5μm in Hematoxylmin Eosin Safran): Peritumoral edema (O), Dermis (D) B. Keratinized cancer cells (x40).

The histological analysis was conducted 7 days after A431 cells injection. The tumor was in intimate contact with derma (Fig 1A) and demonstrated widespread architectural disarray, differentiated squamous cells with tonofilament bundles and abundant keratin pearls (Fig 1B). Intracellular bridges were prominent, probably as a result of loss of cohesion between cells. Cells had all major characteristics of malignant epidermoid cells: dysplasia with increased amount of eosinophilic cytoplasm, nuclear atypia with multipolar abnormal mitotic figures and necrosis. Intense inflammatory process with edema was visualized at the tumor periphery. These observations are hallmarks of keratinizing vulvar squamous cell carcinoma.

### Fluorescence spectroscopy of PpIX after topical m-ALA application

Cutaneous side effects (ulceration, erythema) or signs of distress were not observed up to 6 hours after m-ALA application, neither upon light excitation (405 nm). Measurements of PpIX fluorescence intensity were performed directly on adjacent skin and tumor at 3h and 6h after m-ALA application.

**Fig 2:**
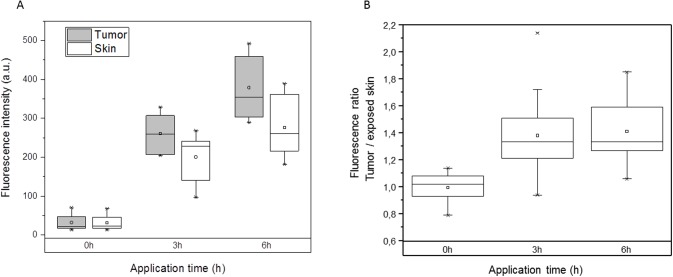
Kinetics of PpIX accumulation in tumor and skin after m-ALA topical application. (A) Box plot showing the evolution of non-invasively assessed fluorescence of tumor and skin as a function of application time (n = 10). The difference in fluorescence values at 3h and 6h compared to T0 was significant (Anova test; p < 0.001). (B) Box plot displaying the fluorescence ratio tumor/adjacent skin after m-ALA application. The difference in ratio at 3h or 6h compared to T0 was significant (Anova test; p < 0.05).

The fluorescence intensity of PpIX in the tumor increased significantly at 3h and 6h (p < 0.001) relative to T0 (Fig 2A). The highest mean of PpIX fluorescence was obtained at 6h after m-ALA application (379.10±83.89 a.u.), being 15.00±5.93 times greater (mean of variations of fluorescence intensity) compared to that before cream application (Table 1). Computed in a similar way, m-ALA application after 3h provided the mean fluorescence of 260.50 a.u. in the tumor, which is 10.82±5.58 times higher compared to T0 (Table 1). It should be noted that the mean autofluorescence was similar in tumor and adjacent skin before m-ALA application (p = 0.640). The fluorescence of unexposed skin was unchanged during the whole observation period (data not shown).

**Table 1:** Non-invasive fluorescence measurements in tissues.

Application time (h)	0h	3h	6h	P_value_			
	Fluorescence (a.u.) Mean ± SD			Global	0 *vs*.3h	0 *vs*. 6h	3 *vs*.6h
Tumor	31.97 ± 20.89	260.50 ± 46.92	379.10 ± 83.89	< 0.001	< 0.001*	< 0.001*	< 0.001
Skin	31.49 ± 18.94	200.51 ± 61.42	276.74 ± 80.97	< 0.001	< 0.001*	< 0.001*	0.043*
Fluorescence ratio	1.00 ± 0.11	1.38 ± 0.35	1.41 ± 0.24	< 0.001	0.021	< 0.001*	1

* p-value < 0.05 with Bonferroni correction

The PpIX fluorescence for m-ALA exposed adjacent skin significantly increased with increasing application time (p < 0.001, Fig 2A). However, the fluorescence values in exposed skin at 3h and 6h were lower than those in the tumor (p = 0.020 at 3h and p<0.001 at 6h, respectively), indicating PpIX selectivity in regard to tumor tissue. As illustrated in Fig 2B, the fluorescence ratio before application was 1±0.11, reflecting similar autofluorescence in tumor and skin. After 3h and 6h of m-ALA topical application, the ratios between tumor and exposed skin were significantly higher than that at 0h (1.38±0.35 at 3h; p = 0.021 and 1.41±0.24 at 6h; p<0,001). The fluorescence ratios between 3h and 6h post m-ALA application were not significantly different (p = 1). The threshold of fluorescence ratio was fixed to 1.2 (mean of autofluorescence + 2SD) and was defined as specific m-ALA induced PpIX fluorescence. This statistic approach is consistent with that reported earlier by Peng *et al*. [16] and was further validated by us using sequence analysis.

### PpIX accumulation at different depths of the tumor

**Fig 3:**
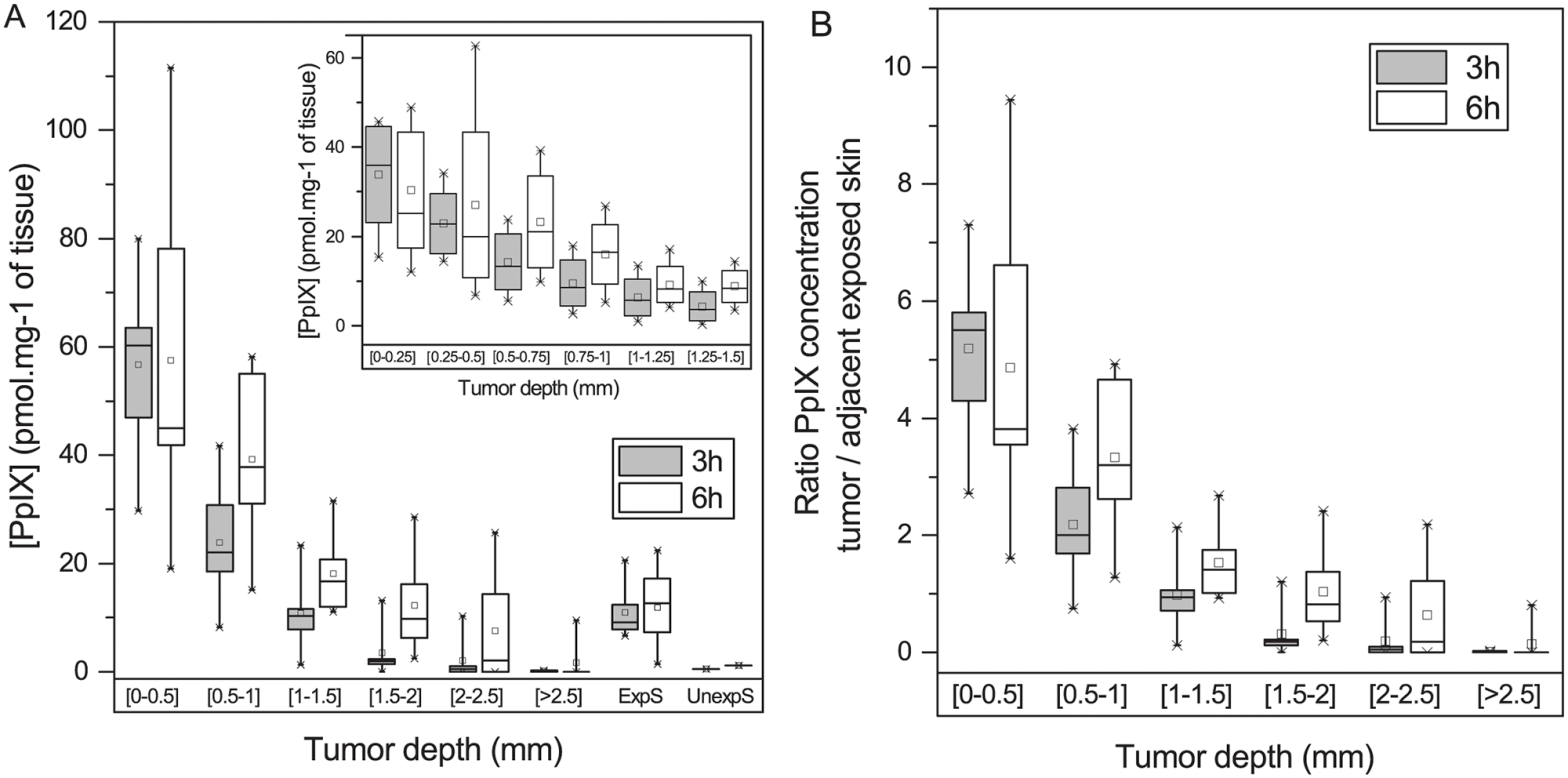
PpIX accumulation at different depths of the tumor after 3h and 6h of topical m-ALA application. (A) m-ALA induced PpIX formation throughout the depth of tumor, assessed every 500 μm up to 2 mm. PpIX accumulation in exposed skin and unexposed adjacent skin is also presented for comparison. Insert to the panel A provides PpIX concentration decay in most superficial tumor region (up to 1.5 mm), measured every 250 μm. (B) PpIX concentration ratio tumor/ adjacent skin at different depth of the tumor after 3h or 6h m-ALA topical application. (ExpS: Exposed skin; UnexpS: Unexposed skin).

PpIX intratumoral distribution throughout the depth of tumor was investigated by spectrofluorescence after PpIX chemical extraction from 250 μm tumor sections (Fig 3A). At the surface of the tumor (0–0.5mm), irrespective of application time, m-ALA induced PpIX formation with high selectivity (≥ 50 pmol.mg^-1^ in tumor tissue *vs* 11 pmol.mg^-1^ in exposed adjacent skin; p = 0.958; Table 2). Important observation is that in superficial tumor area (up to 1.5 mm) PpIX fluorescence tends to show a drastic decrease, especially after 3h of m-ALA application (Fig 3A, insert). Unexposed adjacent skin contained extremely low level of PpIX, close to zero, indicating that m-ALA did not diffuse significantly outside the area of application, whatever the application time (0.48±0.15 and 1.15±2.03 pmol.mg^-1^ of tissue, respectively after 3 and 6h; Table 2).

**Table 2:** Intratissular PpIX concentrations as a function of tumor depth after m-ALA topical application.

Application time (h)	3h (n = 6)	6h (n = 9)	P_value_
	Mean [PpIX] ± SD (pmol.mg^-1^ of tissue)		3 *vs*. 6h
**Tumor depth (mm)**			
[0–0.5]	56.80 ± 16.98	57.48 ± 28.56	0.958
[0.5–1]	23.87 ± 11.39	39.32 ± 14.79	0.034
[1–1.5]	10.77 ± 7.2	18.16 ± 7.00	0.051
[1.5–2]	3.47 ± 4.85	12.23 ± 8.50	0.026
[2–2.5]	2.06 ± 4.06	7.55 ± 9.78	0.201
>2.5	0.11 ± 0.15	1.70 ± 3.51	0.539
**Adjacent skin**			
Exposed skin	10.95 ± 5.15	11.81 ± 6.57	0.814
Unexposed skin	0.48 ± 0.15	1.15 ± 2.03	0.814

Passing deeper into the tumor, the concentration of PpIX decreased progressively at both application times (p<0.001), though higher PpIX values were always observed at 6h (p = 0.020). Between 1.5 and 2.5 mm of tumor depth, the PpIX concentrations were extremely low after 3h of m-ALA application (< 3.5 pmol.mg^-1^ of tissue, Table 2) contrary to 6h where the concentrations of PpIX were stable (≥ 10 pmol.mg^-1^ of tissue). Thus at this time point (6h) the PpIX concentration was 3.5 times higher compared with that at 3h. PpIX was always detectable up to 2.5 mm, the distance corresponding to more than three quarters of the tumor.

The PpIX fluoescence ratios tumor/adjacent exposed skin after m-ALA topical application are shown on the Fig 3B and Table 3. Both time intervals provided a good selectivity up to 1mm depth with the fluorescence ratios exceeding the threshold of 1.2. Moreover, at 6h post-application, the ratio was still 1.54±0.59 up to 1.5mm.

**Table 3:** Fluorescence ratio as a function of tumor depth.

Application time (h)	3h (n = 6)	6h (n = 9)	P_value_
	Fluorescence ratio; Mean ± SD		3 *vs*. 6h
**Tumor depth (mm)**			
[0–0.5]	5.19 ± 1.55	4.87 ± 2.42	0.776
[0.5–1]	2.18 ± 1.04	3.33 ± 1.25	0.068
[1–1.5]	0.98 ± 0.66	1.54 ± 0.59	0.096
[1.5–2]	0.32 ± 0.44	1.04 ± 0.72	0.033
[2–2.5]	0.19 ± 0.37	0.64 ± 0.83	0.218
>2.5	0.01 ± 0.01	0.14 ± 0.30	0.542

## Discussion

Given the potential evolution of vulvar HSIL, its management represents a current diagnostic and therapeutic challenge. Vulvar HSIL must be detected early and addressed correctly for safe and efficacious treatment. Detection is limited to visual assessment with confirmation by histopathology when needed [1]. Colposcopy, or other forms of magnification of the vulva, could be useful but vulvar HSIL aspect varies and it is usually disturbed by edema, erythema, transformations associated with vulvar chronic pruritus or former scar. Thus, the diagnosis is based on the biopsy and the clinician expertise.

Recently fluorescence-assessed photodiagnosis was proposed providing the surgeon with an excellent possibility to enhance visualization of the tumor and its limit, thus improving tumor control [17]. The conversion of ALA or its esterified derivatives to PpIX is favored in malignant and premalignant cells making ALA an excellent targeted probe [18]. These molecules have a leading position in fluorescence-assessed mapping of brain and bladder tumors [7, 19]. m-ALA application also allowed a theranostic approach, namely fluorescence-assessed diagnosis followed by PDT in cutaneous carcinomas [20, 21]. The present study is the first to demonstrate the successful application of m-ALA-PpIX based PDD for vulvar cancer in pre-clinical models.

Firstly, we initiated a murine model of xenografted heterotopic vulvar tumor, using long-established vulvar carcinoma cell line A431. This cell line was characterized in early 70^s^ [22] and was intensively used in successive studies as a keratin-rich epithelial vulvar carcinoma cell line [23, 24]. Indeed, the developed tumor presented all major characteristics of keratinizing vulvar squamous cell carcinoma (Fig 1). This cell line was also used as xenografted model of squamous cells carcinoma of the vulvar [25, 26]. A major weakness of this model is that the most superficial sections (up to 0.5 mm) consisted in the succession of mouse dermis and vulvar tumor, thus remaining remote from vulvar lesions in situ. The most relevant model should be one induced orthotopically, however these tumor models encounter certain complexities in development process. Only one report attempted to develop chemically induced orthotopic vulvar murine cancer [27].

The fluorescence induced by m-ALA application on tumor and adjacent skin was evaluated applying two procedures: direct measurement using fluorescence spectroscopy and quantification of PpIX concentration as a function of the depth of frozen tissues. Of note, the fluorescence of nonexposed adjacent skin did not increase significantly during the procedure, indicating a low diffusion of m-ALA into systemic circulation. This observation is consistent with previous studies [12, 13, 28] showing that a topical m-ALA application is the best drug administration route to provide a strong PpIX fluorescence localization pattern. Unlike m-ALA, 5-ALA diffuses beyond the cutaneous area of interest and enters more readily the circulation [29, 30]. Therefore, the management of m-ALA by the cells would prevent its systemic passage, confining the molecule to the exposed areas. This feature would significantly limit potential side effects in clinical settings.

Our study clearly demonstrated the selective accumulation of photosensitizer in the tumor compared to adjacent tissue from as early as 3h post-mALA application. Indeed, the non-invasive measurements evidenced the higher fluorescence intensity in the tumor compared to adjacent skin, yielding ratios of 1.38 and 1.41 after 3h and 6h applications respectively (Fig 3). These data are consistent with a previous study that reported a ratio of 1.6 after a 6h m-ALA topical application on xenografted colon cancer cells [31]. In clinical settings, the fluorescence induced by m-ALA on basal cell carcinoma was 1.17–1.38 times higher than that on adjacent skin [16].

Despite a linear increase of fluorescence in tumor and adjacent exposed skin as a function of application time, the fluorescence ratios remained fairly stable over time (Fig 2A and 2B). Thus, it is not certain that increasing exposure time will improve the fluorescence-assessed visibility of the lesions. Based on box-plot presentation (Fig 2A), we also observe the increase in fluorescence intensity variability on pathological site and adjacent skin with time. This variability could be related to the heterogeneity of m-ALA penetration into the tumor related to several factors such as skin thickness, depth of the lesion and presence of inflammation or edema.

m-ALA is sufficiently absorbed by the tumor to induce elevated intratumor PpIX levels beyond the surface layers. PpIX extracted from frozen tissues was always detectable up to 2.5 mm, the distance corresponding to more than three quarters of the tumor. Three or six hours intervals demonstrated a very good selectivity with a ratio above 2.0–3.0 at the depth 0.5–1 mm (Fig 3B).

In clinical settings, vulvar HSIL presents mitotic figures, apoptotic cells, and dysmaturation involving greater than one-third of the epithelial thickness (with blocklike p16 staining, a surrogate for HPV infection). In non–hair-bearing human skin, a 1 mm depth is sufficient to explore HSIL. Our results suggest the high potential of topical m-ALA for the diagnosis of vulvar HSIL since these lesions develop on a surface area preserving the basement membrane. However, when HSIL extends down adnexa in hair-bearing skin, HSIL may not be adequately diagnosed with m–ALA based PDD.

Recently published guidelines on the management of vulvar intraepithelial neoplasia indicated the photodynamic therapy as a potential medical treatment for these lesions. Therefore, fluorescence-assessed PDD followed by PDT paves the way for a phototheranostic approach in HSIL management [1, 2].

To summarize, photodynamic diagnosis using in vivo topical m-ALA appears to be a promising way to detect vulvar HSIL. In conclusion, the results of this study could incite future clinical trials for the recognition of high-grade squamous intraepithelial lesion of the vulva. In these clinical trials, a minispectrometer with handheld probe could also facilitate fluorescence measurements with a “no touch” mode [32].
